# Teamwork as an Interprofessional Competency for Collaborative Hospital Practice

**DOI:** 10.3390/nursrep16030082

**Published:** 2026-02-26

**Authors:** Laura Andrian Leal, Ivaneia Alves Pereira Sobrinho, Luan Gagossian Savóia, José Carlos Carvalho, Fabiana Faleiros, Silvia Helena Henriques

**Affiliations:** 1 University of São Paulo at Ribeirão Preto College of Nursing, São Paulo 14040-902, Brazil; fabifaleiros@eerp.usp.br (F.F.); shcamelo@eerp.usp.br (S.H.H.); 2 Interunit Doctoral Program in Nursing, University of São Paulo at Ribeirão Preto College of Nursing, São Paulo 14040-902, Brazil; ivaalves@usp.br; 3 Fundamental Nursing Program, University of São Paulo at Ribeirão Preto College of Nursing, São Paulo 14040-902, Brazil; luansavoia@usp.br; 4 Nursing School, University of Porto, 4200-072 Porto, Portugal; zecarlos@esenf.pt

**Keywords:** professional competence, interdisciplinary practices, interprofessional relationships, health team, Intensive Care Units, hospitals, nursing

## Abstract

**Background/Objectives**: This study aimed to analyze the perceptions and experiences of health professionals regarding teamwork as an interprofessional competency within the context of Intensive Care Units (ICUs) in a Brazilian public teaching hospital. **Methods**: This was a qualitative, exploratory study guided by a constructivist–interpretative perspective. The scenario consisted of Intensive Care Units of a public teaching hospital, which is a reference for emergency care, located in Brazil. Sampling was intentional and involved 29 professionals, most of whom, 25 (86.20%), were females, including nurses, nursing technicians, physicians, physiotherapists, and others. In order to collect data, individual semi-structured face-to-face interviews were conducted in 2025, which were audio-recorded and fully transcribed. The criterion for determining the number of participants was theoretical saturation. Data analysis followed the steps of Braun and Clarke’s thematic analysis, conducted inductively, with peer validation and the use of illustrative quotations to ensure credibility. **Results**: Five main categories emerged: “Understanding teamwork as an interprofessional competency,” “Factors that facilitate interprofessional teamwork,” “Factors that hinder teamwork,” “Tools used in the ICU to develop interprofessional teamwork” and “Individual actions to develop interprofessional teamwork.” The analysis revealed a central tension: although professionals discursively value interprofessional teamwork, its practical implementation is constrained by organizational and hierarchical barriers. Communication was identified as a transversal axis, functioning at times as a facilitator and at other times as a barrier. **Conclusions**: This study demonstrates that interprofessionality in Brazilian ICUs cannot be sustained solely through individual initiatives, but requires structured institutional strategies, such as formal collaboration protocols, interprofessional education programs, and a revision of hospital organizational culture. Furthermore, although health professionals value interprofessional teamwork, their practice still faces significant barriers. These findings may support managers’ reflection on the need to implement in-service teaching and learning strategies that facilitate interprofessional teamwork, especially those in high-technology units, thus enhancing collaborative practice in health.

## 1. Introduction

In healthcare services, such as hospitals, there are highly technology-intensive units known as Intensive Care Units (ICUs). Within this context, the dynamics of the care process rely on qualified professionals, making it essential to foster the training of human resources in health through an integrative approach that combines diverse forms of knowledge and promotes interprofessional teamwork. These aspects are also aligned with the proposal of the Competences in Health Promotion Project (CompHP), which brings together a set of core competencies for interprofessional practice, among which interprofessional teamwork is a central element [1]. Therefore, it is imperative to have the ability to think critically and collaboratively for the provision of excellent care, meaning that it is essential for people to possess so-called interprofessional competencies [2].

In this approach, interprofessional competencies are skills, knowledge, and attitudes that enable effective collaboration among professionals from different fields, with the objective of providing person-centered care or services in an integrated and coordinated manner, that is, fostering interprofessional work [3].

Interprofessional work emerges as one of the main forms of organization for developing favorable and effective perceptions in work relationships. It involves working together with interdependence of actions through the sharing of knowledge, decisions, information, interventions, and empowerment, as well as respect and recognition of one another in the pursuit of common objectives [4,5]. Accordingly, interprofessional work leads to collaborative practices that allow for more effective, comprehensive, and sustainable results than would be possible with individual or isolated work [3].

In this line of reasoning, interprofessional teamwork assumes particular relevance within the context of ICUs, environments characterized by high technological density and rapid decision-making, where diverse professional categories must act in a coordinated manner to ensure comprehensive and safe care. In such settings, understanding teamwork as an interprofessional competence is essential to address the clinical and organizational complexity inherent to the care of critically ill patients.

In this context, an interprofessional competency that is necessary in health services is teamwork, which arises as a reciprocal, two-way relationship between work and the interaction of professionals with health services, aiming for the comprehensive health of patients. In this context, teamwork is defined as a work organization strategy that coordinates actions and different professional knowledge, resulting in comprehensive and quality care [5,6]. In this scenario, the nurse is considered one of the main individuals responsible for coordinating the work of the multidisciplinary team through open and transparent communication [7].

The theoretical framework adopted in this study was the Assessment of Interprofessional Team Collaboration Scale (AITCS II-BR), employed not as a measurement instrument but rather as an analytical reference to guide the interpretation of the dimensions of partnership, cooperation, and coordination present in interprofessional practices. This choice enabled the structuring of the analysis around categories that reflect the relational and organizational dynamics of Intensive Care Units (ICUs), without any intention of quantification [8].

However, it is worth highlighting the scarcity of studies in which the authors investigated teamwork as an interprofessional competency for collaborative practice in health in the ICU setting, which also justifies conducting this study. The literature provides evidence indicating the predominance of international studies focused on strategies for developing interprofessional work aligned with specific themes, such as patient safety or women’s health [9,10,11,12,13].

Despite advances in interprofessional education policies, the national literature still reveals specific gaps regarding how teamwork is understood and experienced in Brazilian ICUs. International studies tend to focus on areas such as women’s health or oncology, yet they seldom explore the institutional and hierarchical barriers that constrain collaborative practice in highly complex environments. This lack of qualitative investigations within the Brazilian context underscores the relevance of the present study. Accordingly, considering the complexity of the work performed by each professional category both individually and collectively within high-complexity units such as ICUs, the severity of the patients treated, and the necessity of employing teamwork competence in favor of interprofessionality, the following guiding question is posed: how do healthcare professionals perceive and experience teamwork as an interprofessional competence in Brazilian hospital ICUs?

Identifying how teamwork is established as a competency for interprofessional work in high-complexity units, such as the ICU setting, the tools used for this work, and the barriers and facilitators in this process can benefit managers and training centers in implementing pedagogical strategies to enhance the development of care, which, consequently, will strengthen comprehensive and safe patient care by enabling interprofessional work in this scenario.

The study aimed to analyze the perceptions and experiences of health professionals regarding teamwork as an interprofessional competency within the context of ICUs in a Brazilian public teaching hospital.

## 2. Materials and Methods

### 2.1. Research Design

This is a qualitative exploratory study, guided by a constructivist–interpretive perspective. The chosen design was deemed appropriate as it enables a deeper understanding of complex and underexplored phenomena, such as interprofessional teamwork in ICUs. This approach is essential for deepening the understanding of little-known phenomena, allowing the generation of hypotheses and the development of new research lines, especially in social and health contexts [14]. The article was prepared in accordance with the guidelines of the Consolidated Criteria for Reporting Qualitative Research (COREQ) [15].

### 2.2. Study Setting

The field of development for this investigation was a public teaching hospital, a reference in emergency and urgent care, located in the Southeast Region of Brazil. The selected hospital is a reference within the Brazilian Unified Health System at the municipal, state, and national levels, providing assistance to patients who require high-technology medical care, such as tissue transplants like liver and kidney transplants, among others. Furthermore, this hospital is a teaching hospital that has a partnership with the higher education institution where the lead researcher works, offering various clinical internship opportunities for undergraduate students.

In order to conduct the research, three ICUs were selected, these being two units for adult patients and one unit for pediatric patients. The choice of the ICUs was due to the fact that they are environments where intense interaction occurs among various professional categories, such as doctors, nurses, physiotherapists, nutritionists, and social workers, all working together to provide integrated care to service users. Moreover, in the ICU setting, it is evident that a patient’s condition can change rapidly, and decisions need to be made quickly and coordinately. In this scenario, interprofessional work becomes not just desirable, but essential to ensure quality care and patient safety.

### 2.3. Population and Sample

The sample was purposive, including the entire healthcare team working in the selected units; that is, nurses, nursing technicians, doctors, physiotherapists, occupational therapists, speech therapists, nutritionists, and psychologists were invited.

### 2.4. Eligibility Criteria

As an eligibility criterion, for all professional categories, participants were considered if they had at least one year of hospital work experience in the institution, taking into account that the professionals had adapted to the operational dynamics of the ICU during this period. Accordingly, it is highlighted that at least two representatives from each professional category were selected to generate substantial data on the topic under investigation. The sampling strategy was purposive and based on convenience, aiming to ensure diversity of professional categories and experiences.

As an exclusion criterion, participants who were on leave, on vacation, absent, or not permanently assigned to the ICU were considered.

### 2.5. Data Collection

For data collection, individual semi-structured interviews were conducted, with an average duration of 20 minutes, with the script developed by one of the authors herself, based on her experiences and on the theoretical framework regarding teamwork as an interprofessional competency [8]. Although the duration was relatively short, efforts were made to ensure depth through open and exploratory questions, validated by three experts in the field. The interviews were audio-recorded and fully transcribed. The script was validated by three experts in the field of interprofessionality and consisted of two parts: The first contains a questionnaire on the participants’ sociodemographic identification data, such as gender, background, years of professional experience, time working in the ICU, and *lato* and *stricto sensu* specialization. The second part contains guiding questions such as the following: What is your understanding of teamwork as an interprofessional competency? Considering interprofessional work, what are the factors that can facilitate or hinder teamwork here in the unit? Are there any tools used to develop teamwork in your unit? Please, provide examples; does the institution promote strategies for developing teamwork aimed at interprofessionality? And you? What have you been doing to improve your teamwork competence at work?

The interviews were conducted by the first researcher and took place individually during the professionals’ break times, in the work environment, according to the participants’ availability in terms of date and time. These interviews took place from May to August 2025 in a room provided by the hospital to ensure privacy.

The number of participants and the number of interviews were determined based on the point of information power, through the simultaneous process of data collection and analysis throughout the development of the research, with the data collection ending as defined by the researcher, taking into account the achievement of the study’s objective, that is, by theoretical saturation [16]. Theoretical saturation was considered reached at the 28th interview, when no new relevant codes emerged. To confirm this, an additional interview (29th) was conducted, which corroborated the absence of new analytical elements.

Before the start of each participant’s interview, an explanation was given about the guarantee of anonymity, seeking to alleviate concerns about any future exposure. Furthermore, the interviewees were informed about the confidentiality of their responses. The interviews were audio-recorded and later manually transcribed, without the aid of software.

### 2.6. Reliability, Authenticity, and Data Analysis

Strategies of credibility and authenticity were adopted, such as validation of the interview guide by experts, triangulation of categories among researchers, and the use of illustrative quotations to support the findings. Reflexivity was taken into account, with the researcher’s impressions recorded during data collection and analysis. The entire set of data was examined according to Braun and Clarke’s (2021) [17] inductive thematic analysis.

The analysis was carried out manually, without the aid of software and independent coding. Thus, the phases of data interpretation and description in this study were considered, such as transcription of data from different data sources into a Word file and familiarization with the data, thus generating initial codes, searching for themes, reviewing themes, defining and naming themes, and producing the analysis report [17].

The researcher’s reflexivity was documented in a field diary and considered in the interpretation of the data, acknowledging its influence on the analytical process.

The study followed the guidelines that regulate the standards for conducting research involving human beings and obtained authorization from the Research Ethics Committee of the proposing institutions, according to Opinion number 7,451,718. It is important to emphasize that the participants were identified by the letter P for participant and were assigned a sequential Arabic numeral, thus ensuring the anonymity of their statements.

## 3. Results

A total of 29 healthcare professionals working in the three selected ICUs (adult and pediatric) participated in the study. There was a predominance of women (86.20%) and a majority held lato sensu specialization in intensive care (51.72%). The duration of professional experience ranged from 1 to 27 years, with a mean of 10 years. Moreover, it is observed that among the participants, only two (6.89%) hold a doctoral degree and four (13.79%) a master’s degree.

It is noteworthy that eight (27.58%) of the total group of interviewed professionals belonged to the pediatric ICU, consisting of: two nurses, one physiotherapist, one speech therapist, two doctors, one occupational therapist, and one psychologist. Moreover, 21 (72.41%) were from the adult ICUs, consisting of six nurses, one clerk, three physiotherapists, two speech therapists, three doctors, one nutritionist, one psychologist, and two occupational therapists. It is emphasized that there was only one nutritionist serving all the investigated ICUs.

Few participants, six (20.68%) out of the total of 29, have a *stricto sensu* graduate degree. Table 1 displays the sociodemographic information.

The thematic analysis generated five main categories, articulated through the theoretical framework of the Interprofessional Team Collaboration Scale (AITCS II-BR), which considers partnership, cooperation, and coordination as central dimensions of interprofessional teamwork competence. Regarding the collected data, the research gave rise to five main categories: “Understanding teamwork as an interprofessional competency,” “Factors that facilitate interprofessional teamwork,” “Factors that hinder teamwork,” “Tools used in the ICU to develop interprofessional teamwork” and “Individual actions to develop interprofessional teamwork.”

**Figure 1:**
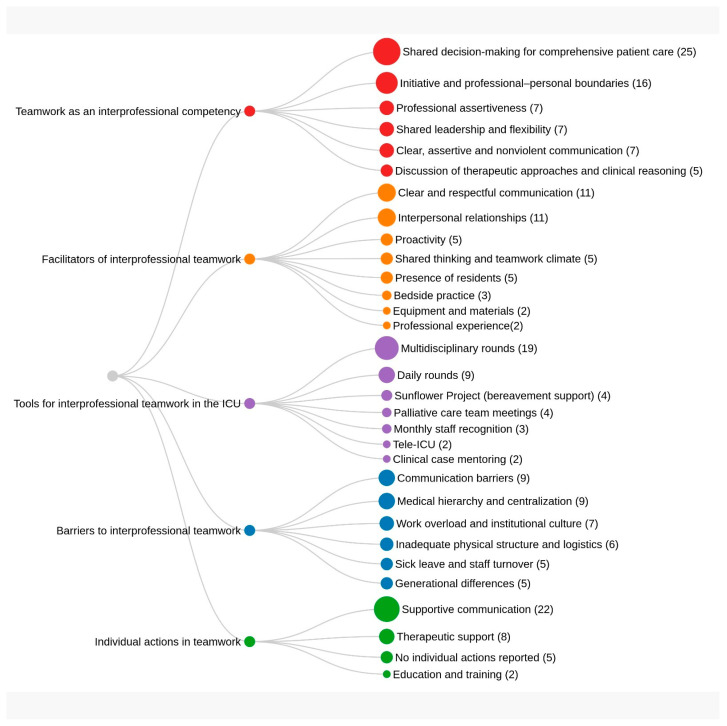
Coding and categorization according to the data extracted from the interviews and associations according to the number of mentions in each interview. Ribeirão Preto, SP, Brazil, 2025.

The categories allowed for the extraction of codings of the main factors associated with each category and their frequency of mention according to the interviews, as displayed in Figure 1.

### 3.1. Understanding Teamwork as an Interprofessional Competence

Participants associated this competence with the process of shared decision-making and the recognition of different professional domains. However, contradictions emerged between discourse and practice:

*“I think that competence in interprofessional teamwork has to do with thinking together, making decisions together, discussing together, aiming for the patient as a whole, you know […]”*

(P11)

*“It has to do with knowing how to position yourself, collaborate, be democratic and have empathy”*

(P7)

*“You should always have clear and respectful communication, non-violent. It’s about knowing how to separate your life from work, you know, and of course understanding and respecting the different areas”*

(P21)

This tension highlights the gap between the ideal of collaboration and the hierarchical reality experienced in the daily routine of ICUs.

### 3.2. Factors That Facilitate Interprofessional Teamwork

Elements such as clear communication, harmonious relationships, and accumulated experience were mentioned. The practice of multiprofessional rounds was valued as a space for exchange and listening. Institutional projects, such as Sunflower (bereavement), as well as the presence of residents and students, expanded critical reflection and fostered cooperation. These findings reinforce the partnership dimension outlined in the theoretical framework, but they also reveal that its effectiveness depends on structural and cultural conditions:

*“Of course, communication is essential for working together like this […]”*

(P1)

*“Without any doubt, having harmony, good relationships, being proactive, and, since we’re in a group, thinking together, are essential”*

(P14)

*“I think some things about the unit also help, such as equipment, bedside care, having students always available […]”*

(P18)

*“First of all, I think professional experience helps; you already have a background, so that you learn how to deal with people and work together”*

(P20)

### 3.3. Factors That Hinder Teamwork

The most recurrent barriers, according to the participants’ perspectives, are associated with medical centralization, work overload and lack of time for meetings, inadequate infrastructure, communication breakdowns, and generational differences.

*“If there is no communication, nothing works, and sometimes speech itself is a barrier; there are people who don’t pass on information, omit it, or don’t know how to communicate […]”*

(P6)

*“Everything is decided by the doctor here; we can’t make decisions on our own.”*

(P9)

*“Because it’s an emergency unit, there’s a lot to do here; so, it’s difficult to sit down together, you want to resolve your own issue and move on to the next one; however, it’s clear that the culture of the doctor like a boss predominates here.”*

(P13)

*“The structure itself doesn’t help. Look! There’s no space for group discussion, besides the fact that a lot of people come and go.”*

(P22)

*“This new generation is complicated, since they don’t want to be collective.”*

(P25)

These factors reveal an overlap between communication problems and hierarchical structures, indicating the need for greater institutional refinement.

### 3.4. Tools Used in the ICU to Develop Interprofessional Teamwork

In addition to multiprofessional rounds, participants highlighted academic mentoring, integration projects, and informal practices of recognition as ways of fostering interprofessional teamwork.

*“Here we have visits, daily team, palliative care […]”*

(P3)

*“The Sunflower project, which is about grief, helps to unite and integrate the team.”*

(P4)

*“Since there are many students, there are tutoring sessions, and that helps.”*

(P15)

*“Besides the visits, I think having the practice of choosing the best employee of the month, bringing cake; that helps to bring people together.”*

(P16)

Although relevant, such tools are informal and do not constitute structured cooperation policies, limiting the scope of the coordination dimension.

### 3.5. Individual Actions to Develop Interprofessional Teamwork

In the absence of institutional training, participants resort to personal strategies such as courses, psychological support, and other initiatives.

*“Since there’s nothing here directed by the hospital, I take public speaking courses or training courses in people management.”*

(P23)

*“I seek individual forms of therapy to gain self-awareness and learn how to deal with the emotions of others, so that we can work better together.”*

(P27)

*“You should be respectful, speak words of comfort to the team”*

(P29)

These initiatives reveal an expanded awareness of the importance of subjectivity in collective work, but they also expose the fragility of relying solely on individual goodwill without organizational support.

## 4. Discussion

Although previous international studies have extensively examined interprofessional teamwork in relation to patient safety, women’s health, and other specific domains, few investigations have qualitatively explored how teamwork is understood and enacted as an interprofessional competency within Brazilian Intensive Care Units. This study contributes new knowledge by situating interprofessional teamwork in the ICU context of a public teaching hospital in Brazil, highlighting the interplay between communication, hierarchical structures, and institutional culture. Unlike prior research that has primarily emphasized structured educational interventions or policy frameworks, our findings reveal the persistence of informal, voluntary, and individually driven strategies to sustain collaboration in high-technology units [18].

By documenting both facilitators (e.g., multiprofessional rounds, academic presence, grief projects) and barriers (e.g., medical centralization, inadequate infrastructure, generational differences), this study expands the existing literature by demonstrating how interprofessional teamwork is shaped not only by professional competencies but also by organizational justice, equity, and the subjective efforts of individual practitioners. In doing so, it advances the understanding of interprofessional practice in critical care settings and underscores the urgent need for institutionalized strategies that move beyond reliance on personal goodwill to achieve sustainable collaborative care.

The analysis of sociodemographic and thematic data from this research reveals significant complexity in understanding and practicing interprofessional teamwork in the studied ICUs. The predominance of female professionals with *latu sensu* training in intensive care underscores a highly specialized profile, but one that still presents limitations regarding *stricto sensu* qualification, which may influence the participation of these professionals in research, teaching, and innovation processes in the field of interprofessionality. The diversity of professional categories, including nurses, nursing technicians, physiotherapists, doctors, speech therapists, occupational therapists, psychologists, and others, reinforces the need for a care model that goes beyond multiprofessional work and achieves true interprofessional collaboration based on shared decision-making and attentive listening [18].

This study confirms findings already described in the international literature regarding the relevance of communication, cooperation, and coordination for interprofessional work in healthcare. However, by situating the analysis within the context of ICUs in a Brazilian public teaching hospital, new perspectives emerge that broaden the understanding of the phenomenon. Unlike environments where institutional interprofessional policies are more consolidated, here, individual and informal initiatives predominate, revealing an organizational gap that limits the sustainability of collaborative practice.

A critical point that emerged from the data of the current research concerns the institutional gap in formalizing interprofessional work models in the hospital context. Although professionals report occasional experiences of collaboration and spontaneous integration initiatives, there is a lack of structured guidelines, defined organizational workflows, and institutional protocols that systematically guide interprofessional practice in the studied ICUs. Such absence ends up restricting interprofessionality to a relational and voluntary dimension, limiting its transformative potential in caring for critically ill patients [19,20].

Thus, interprofessionality in the hospital setting can no longer be seen as an individual choice or a sporadic practice, but as an ethical and organizational guideline [21]. The consolidation of formal and institutionalized models is essential for care to cease being fragmented and to truly become comprehensive and characterized by joint responsibility, as has already been more consistently sought in Primary Care experiences in Brazil.

Regarding the understanding of interprofessional teamwork as a competency, the data indicate that there is a significant appreciation for joint decision-making in favor of comprehensive care. Most professionals highlighted that therapeutic approaches are discussed within the team, with diverse contributions and active listening among members. Nevertheless, this perception often contradicts practice reality, which is marked by a hierarchy in clinical decisions, primarily concentrated within the medical team [22].

Expressions such as “the final word always belongs to the doctors” and “each to their own,” said by the study participants, illustrate the limitations found in building truly shared care. Empathetic and shared leadership was valued in the interviews, being associated with flexibility and recognition of colleagues’ competencies. However, there is a gap between this idealized appreciation and its daily implementation, especially in the face of rigid institutional structures and established hierarchical practices centered on medical authority [23].

A central aspect identified was the tension between normative discourse and concrete practice. Although professionals value joint decision-making and active listening, in practice, medical hierarchy remains the structuring axis of relationships. This discrepancy should be regarded not merely as a contradiction, but rather as a critical finding, as it demonstrates how collaborative norms can be neutralized by entrenched power structures. The analysis of participants’ accounts suggests that interprofessional work is not limited to technical competencies, but also involves symbolic and organizational disputes that can be interpreted through the lens of the sociology of professions and the organization of healthcare work—fields that discuss medical centrality and mechanisms of professional legitimation.

The data also revealed that clear, assertive, and non-violent communication is a cross-cutting and structuring axis of interprofessional relationships. It appears both as a facilitator, when practiced with active listening and respect, and as a barrier, when marked by noise, omissions, or misinterpretations [24]. Professionals who mentioned good communication practices reported greater fluidity in collective work, while those who experience communication failures associate them with conflicts, rework, and insecurity in providing assistance. A relevant finding was the association between good communication and the ability to separate personal from professional matters, an aspect related to emotional maturity and understanding of the role of each profession in caring for critically ill patients.

In this line of thinking, researchers have observed that communication skills are essential for building professionalism, establishing patient trust, and providing the best care to users. Nonetheless, these skills are often neglected in medical training. This highlights the importance of workshops and continuing education. In this sense, researchers implemented a workshop to promote communication skills and found that this strategy raised awareness about the importance of effective use and improved care itself, as well as teamwork, showing that this approach is useful for interprofessional teams, thus helping in avoiding misunderstandings and minimizing errors [24].

Furthermore, facilitating and hindering aspects of interprofessional teamwork in the investigated ICUs were identified. Among the facilitating factors for interprofessional teamwork, the presence of structured multiprofessional rounds, good interpersonal relationships, and accumulated professional experience stand out. The practice of daily rounds with multiple professionals was recognized as an effective space for exchange, listening, and joint decision-making. Projects such as Sunflower, focused on grief care, and *TeleUTI*, with a technological and educational interface, were mentioned as initiatives that broaden the perspective on comprehensive care and strengthen interprofessionality [25].

It is also important to highlight the contribution of the university body, especially the presence of residents and undergraduate students, as an element that facilitates critical reflection on health work. The interaction between trainees and specialists promotes horizontal learning, the exchange of knowledge, and the questioning of centralized practices. Accordingly, continuing education and academic service are strategic components for consolidating collaborative practices in the ICU setting [26].

Conversely, the factors that hinder interprofessional practice are diverse and structural. Communication barriers continue to be one of the main challenges, often mentioned in connection with medical centralization and a lack of active listening from certain professional categories [27]. Work overload and institutional culture were identified as significant obstacles to collaboration, as they limit time for meetings, clinical discussions, and educational activities. The inadequate physical structure, combined with the poor logistics of some sectors, prevents meetings from taking place in appropriate spaces and reduces opportunities for meaningful interaction among team members [28,29].

Moreover, the results show that barriers such as work overload, inadequate infrastructure, and a fragmented institutional culture not only hinder cooperation but also reinforce reliance on individual strategies (courses, therapy, empathetic attitudes). This predominance of personal solutions, to the detriment of institutional policies, reveals a structural fragility that distinguishes the Brazilian context from more systematized international experiences. Thus, the original contribution of this study lies in demonstrating how the absence of formal protocols and institutional spaces for collaboration transforms teamwork into a voluntary and vulnerable practice.

Another relevant fact is the perception of intergenerational difficulties, especially in dealing with professionals from the so-called “Generation Z,” who, according to some participants, show a lower rate of adherence to the traditional work model, requiring new approaches to management, training, and communication [30]. Therefore, it is important to break away from hierarchical and centralized models, which do not favor interprofessional work, but are better suited to the needs of diverse workers.

Individual actions also reveal an effort to resist and reinvent collaborative practices, albeit in a limited way. References to the use of individual strategies such as public speaking courses, therapy, and conversation as efforts to minimize the negative impacts of poor communication were recurrent, since there is no promotion of training on this theme in the investigated ICUs. These data corroborate the work of other researchers who point out that, although there have been advances, hospitals often do not adequately train for interprofessional work, and structural and cultural barriers still limit collaborative training [31].

Professionals who report investing in their continuous training, seeking therapy, or adopting empathetic and proactive attitudes show an expanded awareness of the role of subjectivity in collective work [32]. Nonetheless, relying solely on these individual actions, without the support of robust institutional strategies, highlights the fragility of policies promoting interprofessionality in the studied ICUs. The lack of formal organizational strategies was predominant among the reports, reinforcing the idea that interprofessionality, although valued in words, still lacks concrete institutionalization. This finding is particularly concerning, as it reveals that the development of collaboration continues to be driven by the goodwill of individuals rather than by structured management guidelines [33].

Another point that deserves emphasis is the generational dimension. Perceptions regarding the lower adherence of younger professionals to the traditional model of collectivity indicate the need to rethink management and training strategies, taking into account new forms of engagement and communication. This contextual specificity suggests that interprofessional policies must be sensitive to cultural transformations and intergenerational dynamics.

Finally, another aspect discussed by the participants was space for voices and listening within teams. While some professionals report feeling heard and valued in clinical decisions, many still perceive significant barriers to expressing their opinions, especially when they belong to categories historically marginalized in decision-making processes, such as nursing. This asymmetry in listening reveals a dimension of power that runs through interprofessional relationships, indicating that mere coexistence between categories does not guarantee effective participation. The formalization of listening spaces, such as meetings with rotating leadership, speaking rounds, and facilitated mediations, can be a viable alternative to ensure that all voices are considered in clinical decisions [27,34]. In this sense, practices based on principles of organizational justice and equity stand out as elements that promote healthy, safe, and collaborative work environments.

An important advancement that this study offers to the scientific field lies in its demonstration that interprofessional teamwork in Brazilian Intensive Care Units is sustained largely through informal, voluntary, and individually driven strategies rather than through structured institutional protocols. This finding expands existing knowledge by revealing that, beyond the professional competencies traditionally emphasized in the international literature, collaborative practice in high-technology hospital environments is also profoundly shaped by organizational culture, hierarchical dynamics, and the subjective efforts of individual practitioners. In doing so, the study underscores the need to move beyond reliance on personal goodwill and to establish formalized, equity-oriented institutional strategies that can ensure sustainable and comprehensive interprofessional collaboration in critical care settings.

### 4.1. Limitations

This study has the limitation of having selected only one hospital institution and specific units, such as ICUs. Therefore, it is expected that future research will expand the scope to include more hospital institutions, as well as other units and field intervention studies, for the training of professionals focused on promoting comprehensive, collaborative, and quality care.

In conclusion, it is important to acknowledge the limitations of the study. The sample, restricted to a single public teaching hospital, may limit the transferability of the findings to other contexts, particularly private or non-university settings. Moreover, the predominance of professionals with lato sensu specialization may have influenced how they understand and report interprofessional work. These limitations, far from invalidating the results, underscore the need for caution in generalization and point to future research that explores diverse settings and professional profiles.

### 4.2. Contributions to Nursing

It is noteworthy that the findings of this research offer important contributions to practice, management, and education in health and nursing, especially regarding performance in complex contexts such as the ICU setting. Although the topic involves various professional categories, it was observed that the centrality of communication, empathetic leadership, and active listening were elements widely highlighted in the participants’ statements, reinforcing the strategic position of nursing in mediating interprofessional relationships. It is well known that nursing accounts for a large portion of human resources allocated to health services and, therefore, constitutes a pillar for collaborative practices that lead to interprofessional work. Accordingly, as a profession that works continuously alongside the patient, nursing occupies a privileged role in coordinating different areas of care, being able to act as a central axis for collaborative practices in the hospital environment.

## 5. Conclusions

This study analyzed teamwork as an interprofessional competence in ICUs of a Brazilian public teaching hospital and offered contributions that go beyond the mere reaffirmation of the importance of communication and collaboration. The findings revealed a central tension between normative discourse, which values joint decision-making and cooperation, and concrete practice, marked by medical centralization, work overload, and organizational barriers. This discrepancy demonstrates that interprofessionalism cannot be reduced to individual competencies, but rather depends on institutional structures capable of systematically and equitably sustaining collaborative practices.

The predominance of individual and informal strategies, such as public speaking courses, therapy, or empathetic attitudes, in the absence of institutional policies distinguishes the Brazilian context from more consolidated international experiences and exposes a structural fragility. This observation reinforces the need for formal protocols, adequate spaces for discussion, and interprofessional education programs specifically designed for ICUs, in order to reduce reliance on personal initiative and ensure the sustainability of collaborative practices. Furthermore, it becomes evident that organizational policies must recognize and value the role of different professions, promoting equity and justice within the hospital environment.

The practical implications of this study point to the urgency of investing in structured interprofessional education, institutional protocols that formalize cooperation among professional categories, and infrastructure that facilitates collective meetings and discussions. From a theoretical perspective, the findings contribute to the understanding of power relations and hierarchy in healthcare work, aligning with frameworks from the sociology of professions and the organization of labor. From a research standpoint, future studies should explore other hospital contexts, including private and non-university institutions, investigate the impact of generational differences on adherence to teamwork, and evaluate management strategies capable of reducing rigid hierarchies and strengthening collaborative practices in highly complex environments.

Thus, the conclusion of this study not only reaffirms the relevance of teamwork as an interprofessional competence but also highlights its contextual specificities and institutional implications, offering concrete recommendations for managers, educators, and researchers. By problematizing the discrepancy between discourse and practice, this study contributes to the construction of more sustainable and equitable models of collaboration in healthcare.

## Figures and Tables

**Table 1:** Distribution of sociodemographic information of health professionals in the selected ICUs, according to professional category, years of professional experience (inner circle) and qualification (lato sensu specialization). Source: own elaboration, 2025.

Variable	Category	n	%
Sex	Female	25	86.2
	Male	4	13.8
Age group (years)	20–29	6	20.7
	30–39	12	41.4
	40–49	7	24.1
	50–59	4	13.8
Professional category	Nurse	8	27.6
	Physician	5	17.2
	Physical Therapist	4	13.8
	Speech–Language Pathologist	3	10.3
	Occupational Therapist	3	10.3
	Psychologist	2	6.9
	Nursing Technician	2	6.9
	Nutritionist	1	3.4
	Clerk	1	3.4
Years of professional experience	Up to 5	11	37.9
	6–10	6	20.7
	11–20	7	24.1
	>20	5	17.2
Specialization	Intensive Care (ICU)	14	48.3
	Pediatric ICU	3	10.3
	Other	8	27.6
	None	4	13.8

## Data Availability

The data was collected by healthcare professionals affiliated with an accredited university. The original contributions presented in this study are included in the article. Further inquiries can be directed to the corresponding authors.

## Public Involvement Statement

The data was collected by professionals working at a hospital partnered with the university. There was no influence of authority over the participants. All ethical principles were guaranteed. There was no public involvement in any aspect of this research.

## Guidelines and Standards Statement

In the preparation of this study, the Consolidated Criteria for Reporting Qualitative Research (COREQ) guide from the Equator Network was used.
